# Significance of concurrent use of weekly cisplatin in carbon‐ion radiotherapy for locally advanced adenocarcinoma of the uterine cervix: A propensity score‐matched analysis

**DOI:** 10.1002/cam4.2784

**Published:** 2019-12-31

**Authors:** Noriyuki Okonogi, Masaru Wakatsuki, Shingo Kato, Hiroto Murata, Hiroki Kiyohara, Kumiko Karasawa, Tatsuya Ohno, Hiroshi Tsuji, Takashi Nakano, Makio Shozu, the Working Group of Gynecological Tumors

**Affiliations:** ^1^ QST Hospital National Institutes for Quantum and Radiological Science and Technology Chiba Japan; ^2^ Department of Radiology Jichi Medical University Shimotsuke Japan; ^3^ Department of Radiation Oncology Saitama Medical University International Medical Center Hidaka Japan; ^4^ Department of Radiation Oncology Gunma University Graduate School of Medicine Maebashi Japan; ^5^ Department of Radiation Oncology Japanese Red Cross Maebashi Hospital Maebashi Japan; ^6^ Department of Radiation Oncology Tokyo Women’s Medical University School of Medicine Tokyo Japan; ^7^ Department of Reproductive Medicine Chiba University Graduate School of Medicine Chiba Japan

**Keywords:** adenocarcinoma, carbon‐ion radiotherapy, cisplatin, concurrent chemoradiotherapy, uterine cervical cancer

## Abstract

**Background:**

Although carbon‐ion radiotherapy (C‐ion RT) with concurrent chemotherapy (chemo‐C‐ion RT) is a promising treatment for adenocarcinoma (AC) of the uterine cervix, its long‐term efficacy remains unclear. We evaluated the long‐term significance of concurrent weekly cisplatin and C‐ion RT for locally advanced AC of the uterine cervix.

**Methods:**

We performed a pooled analysis of patients with stage IIB–IVA AC of the uterine cervix who underwent C‐ion RT alone or chemo‐C‐ion RT between September 2007 and December 2018 at our institution. Patients received 74.4 Gy (relative biological effectiveness) with or without cisplatin (40 mg/m^2^ per week for up to 5 weeks), underwent no prior pelvic RT or systemic therapy, and had a performance status of 0‐2. Propensity score matching was based on the year of diagnosis, regional lymph node metastasis, and stage.

**Results:**

The matched cohort contained 26 patients who underwent C‐ion RT and 26 who underwent chemo‐C‐ion RT. The median age and follow‐up period were 57 (range, 28‐79) years and 34 (range, 2‐126) months, respectively. The 5‐year overall survival rate was significantly better in the chemo‐C‐ion RT group (72%) than in the C‐ion RT group (46%; *P* = .041). The 5‐year distant metastatic‐free rate was also significantly better in the chemo‐C‐ion RT group (66%) than in the C‐ion RT group (41%; *P* = .048). The incidence of grade ≥ 3 late toxicities was comparable between the two groups.

**Conclusions:**

Chemo‐C‐ion RT for locally advanced AC of the uterine cervix is associated with a long‐term survival benefit.

## Graphical abstract

Chemo‐C‐ion radiotherapy improves survival for uterine cervical adenocarcinoma. Chemo‐C‐ion radiotherapy has improved distant metastatic‐free rates. C‐ion radiotherapy with cisplatin is more effective than C‐ion radiotherapy alone.

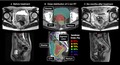

## 1 INTRODUCTION

Uterine cervical cancer continues to affect a sizeable segment of the female population. In 2018, there were 569 000 new cases of uterine cervical cancer worldwide, accounting for 7.5% of all cancer‐related deaths in women reported per annum.^1^ Squamous cell carcinoma (SCC) is the most common histological type, whereas adenocarcinoma (AC) accounts for 10% to 25% of all uterine cervical cancer cases.^2^, ^3^

Radical hysterectomy or definitive radiotherapy (RT) is considered the first‐line treatment for early‐stage uterine cervical cancer, whereas concurrent chemoradiotherapy (CCRT) is the best for locally advanced uterine cervical cancer.^4^, ^5^, ^6^ Although cervical SCC and AC differ in many respects (eg, anatomic origin, risk factors, and rate of metastasis),^7^ current treatment strategies do not distinguish between the two histological types. In fact, previous studies have shown that ACs are more radioresistant than SCCs with poorer outcomes; the 5‐year overall survival (OS) rate for AC is only 0% to 41%.^8^, ^9^, ^10^, ^11^, ^12^

In recent decades, three‐dimensional image‐guided brachytherapy (3D‐IGBT) has become the standard modality for uterine cervical cancer.^13^, ^14^ Many studies have demonstrated its benefits for cervical SCC.^15^, ^16^ However, in several recent studies, it did not appreciably improve the poor local control (LC) or OS rates for AC of the uterine cervix.^17^, ^18^ Therefore, a different approach is warranted for AC of the uterine cervix.

As an alternative approach, we previously examined the effectiveness of carbon‐ion RT (C‐ion RT) for AC of the uterine cervix.^19^, ^20^ We found that 74.4 Gy (relative biological effectiveness [RBE]) in 20 fractions over 5 weeks was tolerable,^19^ with 5‐year OS and LC rates of 38% and 55%, respectively. Notably, this LC rate was comparable to or better than that of previous photon‐based studies without brachytherapy.^8^, ^9^, ^10^, ^11^, ^12^ However, the incidence of distant metastasis (DM) was still high; the 2‐ and 5‐year DM rates were 49% and 65%, respectively.^19^ To reduce the DM rate, we conducted a clinical trial of concurrent use of weekly chemotherapy and C‐ion RT (concurrent chemo‐C‐ion RT) for AC of the uterine cervix.^20^ This study showed the feasibility of weekly administration of cisplatin at 40 mg/m^2^ and C‐ion radiation at 74.4 Gy (RBE) in 20 fractions. The 2‐year OS, LC, and DM rates were 88%, 71%, and 32%, respectively.

Although chemo‐C‐ion RT appears to be a promising treatment strategy for AC of the uterine cervix, its long‐term efficacy remains unclear. The purpose of this study was to compare the long‐term effects of chemo‐C‐ion RT and C‐ion RT alone in patients with locally advanced AC of the uterine cervix via propensity score‐matched analysis.

## 2 MATERIALS AND METHODS

### 2.1 Eligibility criteria

We performed a pooled analysis of data collected from patients treated with C‐ion RT or chemo‐C‐ion RT for uterine AC between September 2007 and December 2018 at our institution. The inclusion criteria were (a) stage IIB, III, or IVA AC of the uterine cervix including the adenosquamous carcinoma subtype, (b) receipt of 74.4 Gy (RBE) of C‐ion radiation with or without weekly cisplatin, (c) no prior pelvic RT or systemic therapy, and (d) an Eastern Cooperative Oncology Group performance status of 0‐2. Staging was performed in accordance with the International Federation of Gynecology and Obstetrics (FIGO) 2008 guidelines. The exclusion criteria were (a) para‐aortic lymph node metastasis on computed tomography (CT) images and (b) severe pelvic infection, psychological illness, diabetes mellitus, or active double cancer. The present study was approved by the Institutional Review Board of the National Institutes for Quantum and Radiological Science and Technology (NIRS: number 19‐007). Participants provided informed consent for participation or had the opportunity to opt‐out of the study. The study was performed in accordance with the principles of the Declaration of Helsinki.

### 2.2 Carbon‐ion radiotherapy and concurrent chemotherapy

The treatment procedures are described in detail in previous reports.^19^, ^20^ A set of 2.5‐ or 5‐mm‐thick CT images was taken for treatment planning, and dose calculation and evaluation were performed using HIPLAN or Xio‐N2 software (National Institute of Radiological Sciences, Chiba, Japan).^21^

Patients received C‐ion RT daily 4 days per week (Tuesday through Friday) for 5 weeks. At each treatment session, the patient was positioned using an orthogonal digital X‐ray positioning system. Throughout the treatment period, patients took laxatives to prevent constipation. To minimize internal motion, 100‐150 mL of normal saline was injected into the bladder. In each treatment session, vaginal packing (ie, cotton pads soaked in contrast medium) was used to allow visualization of the surface of the cervix by an X‐ray during delivery of the last eight fractions.

Gross tumor volume (GTV) was defined by magnetic resonance imaging (MRI) and clinical examinations such as gynecologic examinations. Clinical target volumes (CTVs) consisted of the primary lesion (GTV, uterus, ovaries, parametrium, and upper vagina) and the whole pelvic node region (CTV1), the uterus, parametrium, and swollen pelvic lymph nodes (CTV2), and the whole uterine cervix and GTV (CTV3). All patients underwent planning CT each time the target volume changed. MRI was performed immediately before each planning session for adaptive treatment planning. Details were provided previously.^19^, ^20^

**Figure 1:**
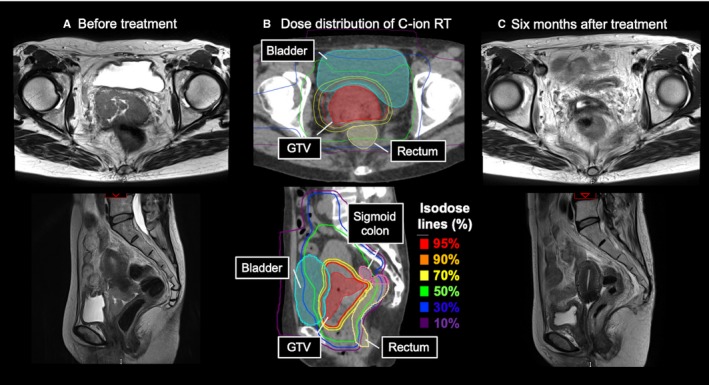
The representative case treated with carbon‐ion radiotherapy. Axial and sagittal MRI T2‐weighted images at before treatment are shown in (A). Dose distributions on CT images are shown in (B). Axial and sagittal MRI T2‐weighted images at 6 months after treatment are shown in (C). Abbreviations: C‐ion RT, carbon‐ion radiotherapy; CT, computed tomography; GTV, gross tumor volume; MRI, magnetic resonance imaging

The initial planning target volume (PTV1) included CTV1 plus a 5‐mm margin for uncertainty and the uterus plus a 10‐mm margin to account for uterine movement during C‐ion RT. PTV2 encompassed CTV2 with a 5‐ to 10‐mm margin. PTV1 and PTV2 were covered by ≥90% of the prescribed dose. PTV3 encompassed the CTV3 with a 3‐mm margin. If the gastrointestinal tract overlapped with PTV3, PTV3 was modified so that the total dose to the gastrointestinal tract would not exceed 60.0 Gy (RBE). The doses for PTV1, PTV2, and PTV3 were 36.0 Gy (RBE) in 12 fractions, 19.2 Gy (RBE) in four fractions, and 19.2 Gy (RBE) in four fractions, respectively. Thus, the total dose to the cervical tumor was 74.4 Gy (RBE) in 20 fractions. The representative case treated with C‐ion RT in Figure 1.

Patients who were ≤70 years old with sufficient kidney function received weekly cisplatin at a dose of 40 mg/m^2^ per week for up to 5 weeks. As in a previous study,^20^ cisplatin administration was discontinued if (a) the patient had a grade ≥3 hematological toxicity, (b) the patient had a grade ≥3 complication in the gastrointestinal tract or urinary system, (c) the serum creatinine value exceeded 1.5 mg/dL, or (d) the aspartate/alanine aminotransferase value exceeded 100 IU/dL.

### 2.3 Assessment of efficacy and toxicity

All patients in this study were followed up every 1‐3 months for the first 2 years and every 3‐6 months thereafter. Recurrences were detected by physical examination, CT, MRI, positron emission tomography, and/or biopsy. Acute toxicity was graded according to the Common Terminology Criteria for Adverse Events (version 4.0).^22^ Late toxicity was graded according to the Radiation Therapy Oncology Group/European Organization for Research and Treatment of Cancer Late Radiation Morbidity Scoring Scheme.^23^

### 2.4 Statistical analyses

To minimize the effect of potential treatment selection bias and confounders on our results, we used propensity score matching to adjust for significant differences in the baseline characteristics of patients. Propensity score matching was accomplished by using 1:1 optimal matching, the nearest neighbor method, and no replacement. Propensity score matching was based on the year of diagnosis, regional lymph node metastasis, and the FIGO stage and was conducted with a stringent caliper of 0.05. LC, OS, and DM‐free rates were calculated by using the Kaplan‐Meier method. Log‐rank, Mann‐Whitney *U*, and chi‐square tests were performed using Statistical Package for the Social Sciences for Macintosh, version 24.0 (IBM Inc, Armonk, NY, USA). A two‐sided *P* < .05 was considered statistically significant in all tests.

## 3 RESULTS

### 3.1 Patient characteristics

The characteristics of the 82 patients who met the eligibility criteria are listed in Supplementary Table 1. Thirty‐seven patients underwent C‐ion RT, and 45 underwent chemo‐C‐ion RT. We used these cohorts for propensity score matching. The matched cohort contained 26 patients who underwent C‐ion RT and 26 who underwent chemo‐C‐ion RT. Among the 52 patients in the matched cohort, the median age and follow‐up period were 57 (range, 28‐79) years and 34 (range, 2‐126) months, respectively. The baseline variables were well matched between the two groups (Table 1).

**Table 1:** Propensity score‐matched patient characteristics

Characteristics	C‐ion RT alone (n = 26)	Chemo‐C‐ion RT (n = 26)	*P*‐value
Year of diagnosis	2007‐2017	2012‐2018	—
Age (median), y	28‐79 (60)	34‐70 (57)	.973
Follow‐up period (median), months	6.6‐125.9 (33.6)	2.4‐76.1 (38.3)	.092
Histology			
Adenocarcinoma	22	23	.685
Adenosquamous carcinoma	4	3	
FIGO stage (2008)			
IIB	12	12	1.000
IIIB	11	11	
IVA	3	3	
Pelvic LN metastasis			
Yes	15	15	1.000
No	11	11	
Tumor size (median), cm	3.1‐8.4 (5.5)	3.0‐12.0 (5.3)	.429
<5 cm	8	11	
≤5 cm to < 7 cm	15	7	
≥7 cm	3	8	
No. of weekly CDDP administrations			
0 times	26	0	<.001
1 time	0	2	
2 times	0	1	
3 times	0	1	
4 times	0	7	
5 times	0	15	

Note

Abbreviations: C‐ion RT: carbon‐ion radiotherapy; CDDP: cisplatin; chemo‐C‐ion RT: carbon‐ion radiotherapy with concurrent chemotherapy; FIGO: International Federation of Gynecology and Obstetrics; LN: lymph node; No: number.

### 3.2 Comparison of treatment efficacy

**Figure 2:**
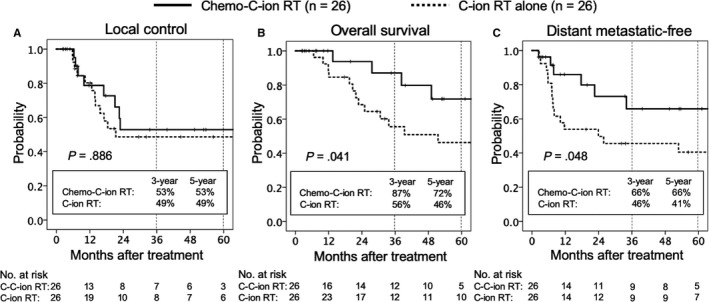
Kaplan‐Meier curves of local control (A), overall survival (B), and distant metastatic‐free rates (C) for all patients analyzed. Solid lines indicate carbon‐ion radiotherapy with concurrent chemotherapy; dashed lines indicate carbon‐ion radiotherapy alone. Number at risk is shown below the figure. Abbreviations: C‐ion RT, carbon‐ion radiotherapy; chemo‐C‐ion RT, carbon‐ion radiotherapy with concurrent chemotherapy

The 3‐year and 5‐year LC rates (95% confidence interval [CI]) were 53% (28.6‐77.2) and 53% (28.6‐77.2) in the chemo‐C‐ion RT group, and 49% (28.0‐69.2) and 49% (28.0‐ 69.2) in the C‐ion RT group, respectively (Figure 2). The differences in the LC rates between the groups were not significant (*P* = .886; Figure 2A). Local recurrences were found in 21 patients by the final follow‐up; 8 and 13 patients in the chemo‐C‐ion RT and C‐ion RT groups, respectively. Among them, 3 of 8 patients in the chemo‐C‐ion RT group and 4 of 13 patients in the C‐ion RT group underwent salvage surgery. The other recurrent patients who did not undergo salvage surgery received systemic chemotherapy. There was no statistical difference in the frequency of salvage surgery between the two groups (*P* = .751).

The 3‐ and 5‐year OS rates (95% CI) were 87% (70.3‐100.0) and 72% (48.1‐95.5) in the chemo‐C‐ion RT group, and 56% (35.8‐75.3) and 46% (26.1‐66.5) in the chemo‐C‐ion group, respectively. The difference in the OS rates between the groups was significant (*P* = .041; Figure 2B).

The 3‐ and 5‐year DM‐free rates (95% CI) were 66% (45.5‐85.1) and 66% (45.5‐85.1) in the chemo‐C‐ion group, and 46% (26.2‐64.9) and 41% (20.9‐60.1) in the C‐ion RT group, respectively. The difference in the DM‐free rates between the groups was significant (*P* = .048; Figure 2C).

**Figure 3:**
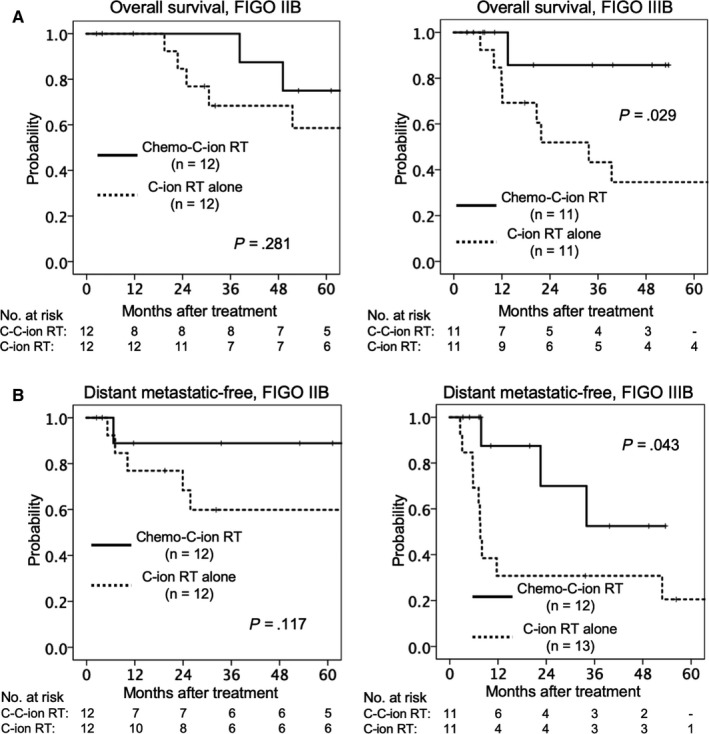
Kaplan‐Meier curves of overall survival (A) and distant metastatic‐free (B) rates according to FIGO stage. Solid lines indicate carbon‐ion radiotherapy with concurrent chemotherapy; dashed lines indicate carbon‐ion radiotherapy alone. Number at risk is shown below the figure. C‐ion RT, carbon‐ion radiotherapy; chemo‐C‐ion RT, carbon‐ion radiotherapy with concurrent chemotherapy; FIGO, International Federation of Gynecology and Obstetrics

Next, we assessed the effects of concurrent chemotherapy on OS and DM‐free rates in FIGO stages IIB and IIIB. FIGO stage IVA was not assessed owing to the small number of stage IVA cases (three per group). For FIGO stage IIB, the OS and DM‐free rates were higher (although not significantly so) in the chemo‐C‐ion RT group than in the C‐ion RT group (Figure 3). However, for FIGO stage IIIB, OS (*P* = .029) and DM‐free (*P* = .043) rates were significantly higher in the chemo‐C‐ion group.

### 3.3 Acute and late toxicities

Regarding acute hematological toxicity, no patients developed grade 4 toxicities in both groups. There was no statistical difference in the incidence of grade ≥3 toxicities between the two groups (Table 2A). Regarding acute nonhematological toxicity, no patients developed grade ≥ 3 toxicities in both groups (Table 2B). In the C‐ion RT group, one patient (4%) had a grade 3 sigmoid colon perforation requiring colostomy 46 months after treatment (Table 2C). In the chemo‐C‐ion RT group, three patients (12%) had grade 3 or 4 toxicities. Among these three patients, one had a grade 4 sigmoid colon perforation requiring colostomy 24 months after treatment. This patient had received repeated laser coagulation surgery for sigmoid colon bleeding before the perforation was detected. The dose to the sigmoid colon was estimated to be 55.2 Gy (RBE). The second patient required salvage surgery for local tumor recurrence 7 months after treatment, as well as colostomy and urinary diversion. The third patient had appendicitis 16 months after treatment and thereafter developed a grade 3 small intestine obstruction that required surgery. The dose to the small intestine was estimated to be <55.2 Gy (RBE). All three of these patients had some pelvic inflammation (eg, repeated laser coagulation, salvage surgery, and appendicitis) after C‐ion RT. These toxicities are described in detail in previous reports.^19^, ^20^

**Table 2:** List of acute and late toxicities

Protocol	No.	Neutrophil decreased grade					Hemoglobin decreased grade					Platelet decreased grade				
		0	1	2	3	4	0	1	2	3	4	0	1	2	3	4
(a) Acute hematological toxicities																
C‐ion RT alone	26	18	3	2	3	0	8	7	9	2	0	26	0	0	0	0
Chemo‐C‐ion RT	26	15	5	5	1	0	2	17	7	0	0	22	2	2	0	0

Protocol	No.	Nausea/vomiting grade					Lower gastrointestinal grade					Genitourinary grade				
		0	1	2	3	4	0	1	2	3	4	0	1	2	3	4
(b) Acute nonhematological toxicities																
C‐ion RT alone	26	20	6	0	0	0	13	11	2	0	0	21	5	0	0	0
Chemo‐C‐ion RT	26	11	10	5	0	0	8	16	2	0	0	23	3	0	0	0

Protocol	No.	Rectum/sigmoid grade					Small intestine grade					Genitourinary grade				
		0	1	2	3	4	0	1	2	3	4	0	1	2	3	4
(c) Late nonhematological toxicities																
C‐ion RT alone	26	15	10	0	1^a^	0	21	3	2	0	0	18	7	1	0	0
Chemo‐C‐ion RT	26	15	6	3	1^b^	1^c^	20	5	0	1^d^	0	18	5	2	1^b^	0

Note

Abbreviations: C‐ion RT: carbon‐ion radiotherapy; chemo‐C‐ion RT: carbon‐ion radiotherapy with concurrent chemotherapy; No.: number.

a Sigmoid colon perforation 46 months after treatment which required a colostomy.

b Salvage surgery for local tumor recurrence 7 months after treatment. This patient required a colostomy and urinary diversion.

c Sigmoid colon perforation 24 months after treatment which required a colostomy. This patient underwent repeated laser coagulation for sigmoid colon bleeding before the perforation was detected.

d Peritonitis caused by appendicitis 16 months after treatment; developed a grade 3 small intestine complication 17 months after treatment.

There was no significant difference in the incidence of grade ≥ 3 toxicities between the two groups (*P* = .602).

## 4 DISCUSSION

This is the first study to evaluate the long‐term efficacy of chemo‐C‐ion RT for locally advanced AC of the uterine cervix. Via propensity score‐matched analysis, we found that concurrent use of cisplatin and C‐ion RT clearly increased OS and DM‐free rates compared with C‐ion RT alone.

As shown in Table 3, previous studies of conventional RT or CCRT reported 5‐year OS rates of up to 41% for locally advanced AC of the uterine cervix.^8^, ^9^, ^10^, ^11^, ^12^ In contrast, the 5‐year OS rate in our study of chemo‐C‐ion RT for locally advanced AC uterine cervix was 72%. Whether CCRT with 3D‐IGBT is an effective strategy for AC of the uterine cervix, as it is for SCC of the uterine cervix,^13^, ^14^, ^15^, ^16^ is unclear owing to the small number of patients (40 of 731 patients) analyzed in a previous study.^16^ In recent reports, uterine ACs had poorer LC and OS rates than did uterine SCCs, even when CCRT with 3D‐IGBT was applied.^17^, ^18^ These findings and those of the present study support chemo‐C‐ion RT as the treatment of choice for locally advanced AC of the uterine cervix.

**Table 3:** Review of previously literature and this study

Author (y)	Stage	No.	Treatment	5y OS (%)	5y LC (%)	Late toxicity ≥ grade 3
Grigsby PW (1988)	III	12	RT	25	33	N/R
Eifel PJ (1990)	III	61	RT	26	46	14%
Lea JS (2002)	III‐IVA	36	RT/CCRT	0	N/R	N/R
Niibe Y (2010)	IIIB	61	RT/CCRT	22	36	N/R
Chen JL (2014)	IIB‐IVA	35	RT/CCRT	41	64	22%
Present study	IIB‐IVA	26	C‐ion RT alone	46	53	4%
		26	Chemo‐C‐ion RT	72	59	12%

Note

Abbreviations: 5y: 5‐year; C‐ion RT: carbon‐ion radiotherapy; CCRT: concurrent chemoradiotherapy; chemo‐C‐ion RT: carbon‐ion radiotherapy with concurrent chemotherapy; LC: local control; N/R: not reported; No.: number; OS: overall survival; RT: radiotherapy.

The present study showed that concurrent use of cisplatin and C‐ion RT improves OS and DM‐free rates compared with C‐ion RT alone. However, unlike a previous study in which conventional CCRT reduced the incidence of both local recurrence and DM compared with RT alone,^24^ it had no effect on the LC rate in our study. The reason for this discrepancy is unclear but may reflect differences in the patient cohorts: in the previous study, patients primarily had SCC of the uterine cervix, whereas our study only included patients with AC of the uterine cervix. Britten et al found that cisplatin acted as a radiosensitizer in only 4 of the 19 human cervical tumor cell lines examined.^25^ This study suggests that radiosensitization by cisplatin depends on the type of cervical tumor cells. The inherent biological nature of C‐ion beams should also be considered.^26^ Although chemotherapeutic drugs have the potential to enhance the tumor cell‐killing activity of carbon ions,^27^, ^28^ Kobayashi et al reported that carbon ions are less sensitive to cisplatin than are X‐rays.^29^ Further characterization of the sensitivity of C‐ions to cisplatin in human cervical cancer cell lines is needed.

In the present study, favorable OS and DM‐free rates were observed, and chemo‐C‐ion RT had a therapeutic advantage over C‐ion RT alone, especially in patients with stage IIIB AC. Salvage surgery may explain the different LC and OS rates in both groups. Overall, however, the 5‐year LC rate was still unsatisfactory. In a recent phase 1 study of C‐ion RT with brachytherapy, five of six patients maintained LC for a median period of 47.5 months.^30^ Although this result is preliminary, it encourages efforts to improve the LC rate in large‐scale studies.

Regarding the incidence of severe acute toxicities, several patients in the chemo‐C‐ion RT group developed grade 2 nausea or thrombocytopenia. However, there was no statistical difference in the incidence of severe toxicities between the two groups. This may be as a result of good dose distribution of C‐ion RT; thereby minimizing the dose to the bone marrow or intestines. The late toxicities in the chemo‐C‐ion RT group in this study were reported previously,^20^ and no additional severe toxicities were observed in this long‐term analysis. The incidence of grade ≥3 toxicities did not differ significantly between the chemo‐C‐ion RT and C‐ion RT groups and was similar to that in previous studies of conventional CCRT (Table 3). These data further highlight the benefits of chemo‐C‐ion RT for AC of the uterine cervix.

As limitations, the present study was retrospective and conducted at a single institution. Moreover, there may have been time‐based differences in patient care.

In conclusion, we demonstrated the long‐term survival benefits of chemo‐C‐ion RT for locally advanced AC of the uterine cervix. Our findings warrant further investigation of the therapeutic efficacy of chemo‐C‐ion RT in a larger number of patients. Although currently limited, the number of facilities providing C‐ion therapy is gradually increasing. Hence, chemo‐C‐ion RT for AC of the uterine cervix will be available in many institutions in the future period.

## CONFLICT OF INTEREST

The authors have no conflict of interest to disclose.
