# Supplementary material for: Significance of concurrent use of weekly cisplatin in carbon‐ion radiotherapy for locally advanced adenocarcinoma of the uterine cervix: A propensity score‐matched analysis
Source: Cancer Med. 2019 Dec 31;9(4):1400–8. doi: 10.1002/cam4.2784 (PMC7013060; doi:10.1002/cam4.2784)
Supplement: Supplementary file 1 [file CAM4-9-1400-s001.docx]

**Supplementary** **Table 1.** Characteristics of 82 patients who met the eligibility criteria

| **Characteristics** | **C-ion RT alone**  **n = 37** | **Chemo-C-ion RT**  **n = 45** | ***p*-value** |
| --- | --- | --- | --- |
| Year of diagnosis | 2007-2018 | 2012-2018 | - |
| Age (median), years | 28-79 (63) | 26-70 (49) | <0.001 |
| Follow-up period (median), months | 2.3-125.8 (33.7) | 2.4-76.1 (39.7) | 0.106 |
| Histology |  |  |  |
| Adenocarcinoma  Adenosquamous carcinoma | 32  5 | 39  6 | 0.981 |
| FIGO stage (2008) |  |  |  |
| IIB  IIIB  IVA | 21  14  2 | 24  18  3 | 0.941 |
| Pelvic LN metastasis |  |  |  |
| Yes  No | 16  21 | 16  29 | 0.629 |
| Tumor size (median), cm | 3.1-12.0 (5.5) | 3.0-12.0 (5.3) | 0.410 |
| <5 cm  ≤5 cm to <7 cm  ≥7 cm | 14  16  7 | 16  15  14 |  |
| No. of weekly CDDP administrations |  |  |  |
| 0 times  1 time  2 times  3 times  4 times  5 times | 37  0  0  0  0  0 | 0  2  1  2  10  30 | <0.001 |

Abbreviations:

C-ion RT: Carbon-ion radiotherapy, Chemo-C-ion RT: Carbon-ion radiotherapy with concurrent chemotherapy, FIGO: International Federation of Gynecology and Obstetrics, LN: Lymph node, CDDP: cisplatin.
